# Fatty acid composition indicating diverse habitat use in coral reef fishes in the Malaysian South China Sea

**DOI:** 10.1186/s40659-015-0004-0

**Published:** 2015-02-22

**Authors:** Takaomi Arai, Razikin Amalina, Zainudin Bachok

**Affiliations:** Institute of Oceanography and Environment, Universiti Malaysia Terengganu, 21030 Kuala Terengganu, Terengganu Malaysia; School of Marine Science and Environment, Universiti Malaysia Terengganu, 21030 Kuala Terengganu, Terengganu Malaysia

**Keywords:** Coral fish, Habitat ecology, Migration, South China Sea, Biochemical signature, Malaysia

## Abstract

**Background:**

In order to understand feeding ecology and habitat use of coral reef fish, fatty acid composition was examined in five coral reef fishes, *Thalassoma lunare*, *Lutjanus lutjanus*, *Abudefduf bengalensis*, *Scarus rivulatus* and *Scolopsis affinis* collected in the Bidong Island of Malaysian South China Sea.

**Results:**

Proportions of saturated fatty acids (SAFA) ranged 57.2% 74.2%, with the highest proportions in fatty acids, the second highest was monounsaturated fatty acids (MUFA) ranged from 21.4% to 39.0% and the proportion of polyunsaturated fatty acids (PUFA) was the lowest ranged from 2.8% to 14.1%. Each fatty acid composition differed among fishes, suggesting diverse feeding ecology, habitat use and migration during the fishes’ life history in the coral reef habitats.

**Conclusions:**

Diets of the coral fish species might vary among species in spite of that each species are living sympatrically. Differences in fatty acid profiles might not just be considered with respect to the diets, but might be based on the habitat and migration.

## Background

Southeast Asia is the global centre for coral reefs, and more than 30% of the Earth’s coral reefs can find in the area [1]. Ecologically, the coral reefs of the South China Sea are sources of larvae and juveniles for many commercially important reef fish. Furthermore, coral reefs are considering important breeding and nursery grounds for many pelagic and demersal fish species found in the open sea [2,3].

Malaysia has the one of the highest and richest diversity of fish in the world [4]. Ambak et al. [5] and Chong et al. [6] listed 2243 and 1951 fish species, respectively, in Malaysian waters. Although several information regarding taxonomy and distribution in coral fish species is available in Malaysian water, few study has done for their life history, ecology and reproduction compared to other coral reef area.

Signature of fatty acid analysis has been increasingly used to study the diet of a number of marine species [7-9]. The use of fatty acids as trophic biomarkers is based on the assumption that many fatty acids in the marine environment are characteristic of specific groups [8]. These fatty acids can generally not be synthesised in higher trophic levels and are incorporated into tissues of higher trophic individuals [10]. Thus, the signature can be useful to trace diet of fish in ecosystem.

In the present study, fatty acid analyses were used to investigate the habitat ecology of five coral reef fish species, *Thalassoma lunare*, *Lutjanus lutjanus*, *Abudefduf bengalensis*, *Scarus rivulatus* and *Scolopsis affinis* collected in the Bidong Island of the Malaysian South China Sea where few information have reported in ecology and biology in the fishes.

## Results

Proportions of saturated fatty acids (SAFA) ranged 57.2% 60.8%, with the highest proportions in fatty acids (Table 1). Palmitic acid (C16:0) was the most common saturated fatty acid ranged from 43.4% to 74.2% (Table 1). Significant differences were found in C14:0 (myristoleic acid), C16:0, C18:0 (stearic acid), C20:0 (arachidic acid) and ∑SAFA between fish species (p < 0.05-0.0001).

**Table 1:** **Fatty acid composition (mean ± SD) in livers of five species of coral fishes collected in the Bidong Island, Malaysian South China Sea**

**Fatty acids**	***Thalassoma lunare***	***Lutjanus lutjanus***	***Abudefduf bengalensis***	***Scarus rivulatus***	***Scolopsis affinis***
	**(n = 6)**	**(n = 6)**	**(n = 5)**	**(n = 5)**	**(n = 3)**
SAFA					
C14:0	14.3 ± 3.8	4.5 ± 0.7	6.0 ± 1.8	10.5 ± 2.0	3.1 ± 0.7
C16:0	44.6 ± 12.4	48.6 ± 8.2	62.4 ± 9.0	43.4 ± 3.3	54.0 ± 3.2
C18:0	0.0 ± 0.0	7.4 ± 4.9	5.6 ± 3.4	5.8 ± 1.6	0.02 ± 0.03
C20:0	0.04 ± 0.06	0.3 ± 0.2	0.1 ± 0.1	0.4 ± 0.3	0.1 ± 0.1
**∑SAFA**	**58.9 ± 10.4**	**60.8 ± 9.2**	**74.2 ± 7.8**	**60.1 ± 3.3**	**57.2 ± 3.6**
MUFA					
C16:1	6.4 ± 2.5	9.6 ± 2.6	2.1 ± 1.7	18.8 ± 3.8	6.9 ± 1.3
C17:1	0.8 ± 0.5	1.4 ± 0.4	1.1 ± 0.5	1.4 ± 0.6	2.2 ± 0.8
C18:1ω9c	11.8 ± 6.6	15.1 ± 6.4	11.6 ± 8.6	5.0 ± 0.9	18.3 ± 10.7
C18:1ω9t	16.0 ± 9.2	9.5 ± 6.0	6.0 ± 6.5	0.4 ± 0.9	11.3 ± 6.9
C20:1	1.8 ± 1.4	0.8 ± 0.4	0.6 ± 0.8	0.2 ± 0.1	0.3 ± 0.2
**∑MUFA**	**36.9 ± 9.2**	**36.4 ± 9.4**	**21.4 ± 8.0**	**25.7 ± 2.8**	**39.0 ± 3.7**
PUFA					
C18:3n3	1.4 ± 0.6	1.1 ± 0.3	1.6 ± 0.8	2.5 ± 0.8	1.2 ± 0.4
C18:3n6	0.4 ± 0.2	0.08 ± 0.09	1.3 ± 0.5	1.4 ± 0.5	0.3 ± 0.2
C20:3n3	0.4 ± 0.3	0.01 ± 0.03	0.2 ± 0.1	4.1 ± 1.0	0.5 ± 0.7
C20:5n3 (EPA)	1.4 ± 1.7	0.6 ± 0.3	1.3 ± 0.9	4.8 ± 2.4	1.0 ± 0.6
C22:6n3 (DHA)	0.6 ± 0.6	1.1 ± 0.8	0.1 ± 0.1	1.4 ± 0.7	0.8 ± 0.8
**∑PUFA**	**4.3 ± 2.8**	**2.8 ± 1.1**	**4.4 ± 1.5**	**14.1 ± 5.2**	**3.8 ± 1.6**

Monounsaturated fatty acids (MUFA) were the second dominant ranged from 21.4% to 39.0% (Table 1). Of all MUFA, oleic acid (C18:1ω9c) was the dominant MUFA for all size classes, followed by C16:1 (palmitoleic acid) and C18:1ω9t (elaidic acid) (Table 1). No significant differences were found between fish species (p > 0.05). Significant differences were found in C16:1, C18:1ω9t and ∑MUFA between fish species (p < 0.05-0.0005), however no significant differences were found in other fatty acids between species (p > 0.05).

The proportion of polyunsaturated fatty acids (PUFA) was accordingly low ranged from 2.8% to 14.1% (Table 1). Linolenic acid (C18:3n3) generally showed highest ranged from 1.1% to 2.5%, followed by EPA (C20:5n3) (Table 1). No significant differences were found between fish species (p > 0.05). Significant differences were found in each SAFA and ∑SAFA between fish species (p < 0.05-0.001). ∑PUFA in *Scarus rivulatus* was the highest proportion between five fish species (p < 0.05-0.01).

Stomach contents were observed for all coral reef fishes. However, stomach content for each fish could not identify prey organisms under macro- and micro-observations.

## Discussion

It is noteworthy that fatty acid composition was different between five coral fish species, although all fishes were collected in same area. Differences in individual fatty acid profiles were reported previously with various factors such as food habits [7-9] and habitat use. However, differences in fatty acid composition in relation to fish species have not been well reported in coral fish species in the area. It is likely that such differences are caused by differences in the diet, behavior and migration of the fish species. Coral fishes were found in coastal and mangrove area during the life [11-13]. The role of mangroves as nursery habitats for some coral fish species has received considerable attention as a link with adjacent coral reef or offshore habitats [14-16]. The diet shifts of coral reef fish species that inhabit mangroves have been reported their early life stages [17-19]. These findings suggest that differences in fatty acid profile between species found in the present study might correspond to the diet and habitat preference in each fish species.

SAFA was the most abundant fatty acids and the palmitic SAFA showed highest values among all fatty acids (Table 1). The second most abundant SAFA was stearic acid. These two SAFAs have been reported to have the highest concentrations in other fish species [20,21]. Fishes from warm waters tended to show high levels of palmitic and stearic acids compared to those from cold waters. This difference is due to metabolic differences between cold and warm water species, because these fatty acids are not usually subject to differences in diet [22]. All coral fishes were collected in the South China Sea in tropical waters, and thus the fish might have higher palmitic and stearic acid levels in the present study.

MUFA was the second most abundant fatty acids, with highest values for oleic acid (Table 1). This is in agreement with findings in copepod [23], Acetes [24] and fish fatty acid profiles [20-22,25]. Oleic MUFA is naturally occurring in large concentrations in many marine organisms, which can also synthesise this MUFA de novo [26]. High proportions of MUFAs of marine predators are generally derived from marine zooplankton [27,28]. In the present study, we did not conduct fatty acid analyses for potential prey organisms. Nevertheless, the higher level of MUFAs found in those coral reef fishes suggest that the fish might feed copepod as one of potential prey organism during the life history.

PUFA composition is generally recognized to vary among fish species. Signature of fatty acids, especially PUFA, is found to derive from the diet as fish has lack of ability to synthesize PUFA [29,30]. *Scarus rivulatus* showed the highest ∑PUFA levels among five coral reef fishes in the present study (Table 1). Like other scarids, the species is herbivores fed on various kinds of microscopic algae on coral skeletons. The diets and feeding behavior are different from other four species studied in the present study. The present PUFA profiles might be reflected feeding ecology and habitat use in the coral reef fishes.

Although stomach content analysis is the basic and conventional approach used to assess fish diet [31], the present study could not observe the prey organisms in stomachs. The analysis has some shortcomings. First, this technique only provides recent feeding and may not accurately reflect the composition of prey items that contribute most significantly to its general diet [9,32]. This technique may also not necessarily account for ontogenetic or seasonal shifts in diet nor regional variability in the diet of a species [9,32]. For a comprehensive understanding of a species’ diet, many specimens must be examined with samples from different seasons, locations, size classes and sexes. Second, stomach content analysis is heavily biased towards items resistant to digestion such as bones, exoskeletons, chelae and eyeballs [33]. Fatty acid signature has been increasingly used to study the diet of a number of marine species. Furthermore, differences in fatty acid profiles might not just be considered with respect to the diets, but might be based on the habitat and migration. Further studies are needed to study for various organisms in coral ecosystem using fatty acid signature for understanding life history and ecology details in the coral fish species.

## Conclusion

Fatty acid signature has been increasingly used to study the diet of a number of marine species. The present study suggests that diets of the coral fish species might vary among species in spite of that each species are living sympatrically in the coral reef habitat. However, we did not examine fatty acid signature for possible prey items in the coral reef habitat. Thus, to elucidate comprehensive food web structure in the habitat, further detail analysis using fatty acid and stable isotope signatures would be needed.

## Methods

### Fish collection

**Figure 1:**
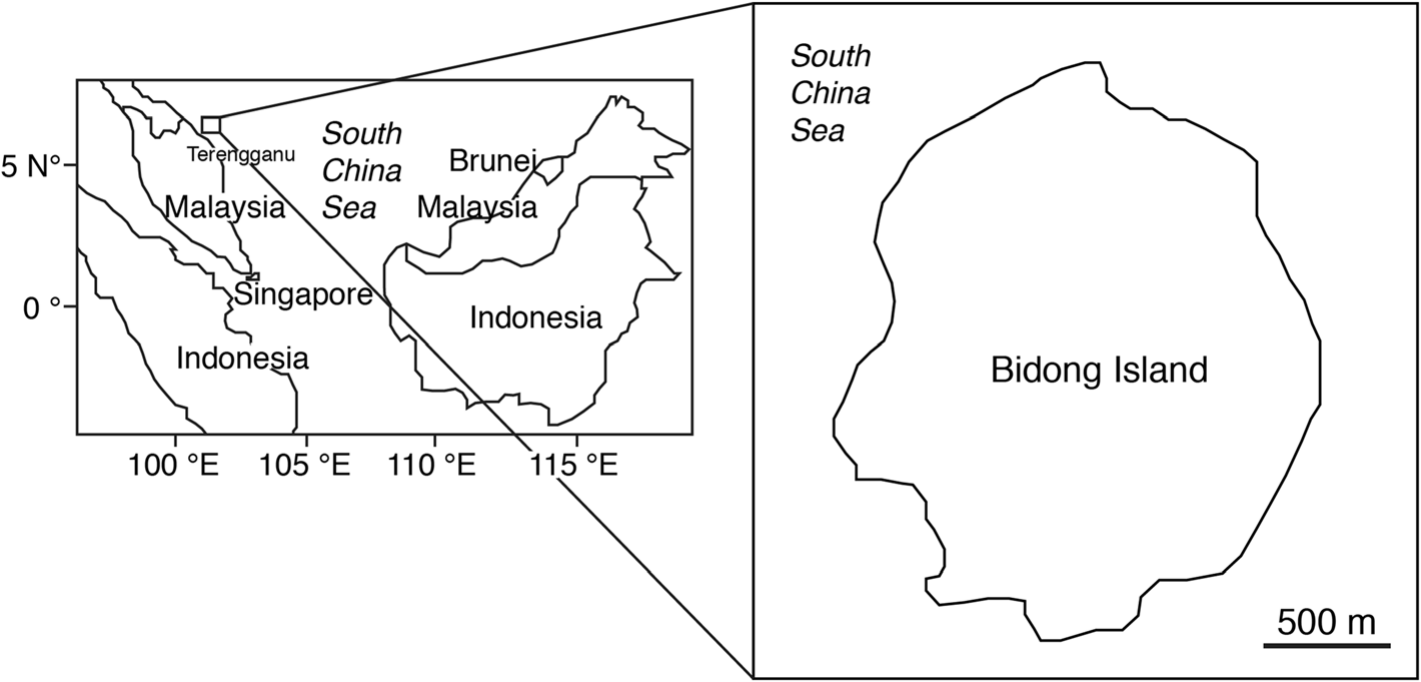
**Map showing the location of the study site at the Bidong Island in Malaysian South China Sea, off the Terengganu State in the east coast of Peninsula Malaysia.**

Five coral reef fish species, *Thalassoma lunare*, *Lutjanus lutjanus*, *Abudefduf bengalensis*, *Scarus rivulatus* and *Scolopsis affinis* were collected at the Bidong Island in the South China Sea, Malaysia (Latitude 5.62°, Longitude 103.07°) between 27 and 28 October 2014 (Figure 1). Bidong Island is located off Terengganu State on the east coast of Peninsular Malaysia, known for its history as Vietnamese refugee settlement. The island also comprises of being well-developed coral reef ecosystems comprising variety of coral and rocky reef associated fishes [13]. All fishes were collected by means of fish traps and hook and line. After collecting, all fishes were immediately stored in ice chest, brought back to laboratory, were kept in −20°C freezer and conducted fatty acid analyses within one month. A total of twenty-five fish samples were measured in total length (TL), body weight (BW), and each fish was dissected, liver and the gonad removed to determine their weights (Table 2). Stomach for each fish was dissected for the content analyses.

**Table 2:** **Biological information of coral fishes collected in the Bidong Island, Malaysian South China Sea**

	**Total length (cm)**		**Body weight (g)**		**Liver weight (g)**		
**Species**	**Mean ± sd**	**Range**	**Mean ± sd**	**Range**	**Mean ± sd**	**Range**	**N**
*Thalassoma lunare*	21.0 ± 2.5	17.6 – 24.2	92.6 ± 26.6	14.1 - 23.3	1.22 ± 0.43	0.76 - 1.92	6
*Lutjanus lutjanus*	18.1 ± 0.8	17.0 - 19.5	79.0 ± 13.3	62.8 - 109	0.51 ± 0.16	0.04 - 0.39	6
*Abudefduf bengalensis*	14.9 ± 0.6	14.3 - 16.0	93.0 ± 16.8	74.6 - 116.9	1.26 ± 0.33	0.92 - 1.62	5
*Scarus rivulatus*	23.0 ± 2.6	20.6 - 26.7	250 ± 88.7	163 - 378	15.4 ± 9.3	3.71 - 28.9	5
*Scolopsis affinis*	19.2 ± 2.0	17.2 - 21.1	106 ± 26.3	77.7 - 130	1.08 ± 0.13	0.95 - 1.21	3

N: total number of specimens.

### Fatty acid analysis

Liver samples of each fish were analysed for fatty acid composition following the one step method [34,35]. Three replicates of each liver and tissue samples were mixed with 4 ml of hexane and 1 ml of internal standard solution in a 50 ml centrifuge tube. After adding 2 ml of 14% BF3 in methanol, the tube was flushed with nitrogen gas. The capped tube was heated on a hot plate at 100°C for 120 min. One ml of hexane was added followed by 2 ml of distilled water. The tube was then shaken vigorously for 1 min and centrifuged for 3 min at 2500 rpm.

Samples were then analysed using a GC-FID (GC 14-B, Shimadzu). Separation was performed with an FFAP-polar capillary column (30 m × 0.32 mm internal diameter, 0.25 μm film thickness). Hydrogen was used as a carrier gas. After injection at 60°C, the oven temperature was raised to 150°C at a rate 40°C min^−1^, then to 230°C at 3°C min^−1^, and finally held constant for 30 min. The flame ionization was held at 240°C. Peaks were identified by comparing their retention times with those of authentic standards (Supelco Inc.). Fatty acids were designated as an n: pωx, where n is the number of carbon atoms in the aliphatic chain, p is the number of double bonds and x is the position of the first double bond from the terminal methyl group. The analytical precision for samples was generally <5% for total amounts and major components.

### Data analyses

Fatty acid concentrations (mg g^−1^ dry weight) were calculated by comparing the peak area of fatty acid in the sample with the peak area of internal standard. The percentage for each fatty acid was converted from the area of chromatogram peaks. The composition is expressed as percentage of total fatty acids (Table 1).

Differences between data were analysed using the Mann–Whitney U-test. Differences among data were also examined using the Kruskal–Wallis test while using the Mann–Whitney U-test for post hoc two-group comparisons. The significance of the correlation coefficient and the regression slope were determined using a t-test (Sokal and Rohlf) [36].

### Ethical standards

This study has been conducted in a field station at the Bidong Island belonging to Universiti Malaysia Terengganu. This study was also reviewed and approved by the Universiti Malaysia Terengganu ethics board.
